# Acute stroke-like deficits associated with nonketotic hyperglycemic hyperosmolar state: an illustrative case and systematic review of literature

**DOI:** 10.1007/s10072-022-06088-7

**Published:** 2022-04-28

**Authors:** Simone Rossi, Michele Romoli, Giacomo Urbinati, Matteo Benini, Michele Russo, Lucio D’Anna, Samir Abu-Rumeileh, Simona Sacco, Pietro Querzani, Matteo Foschi

**Affiliations:** 1 IRCCS Istituto delle Scienze Neurologiche di Bologna, Bologna, Italy; 2 Department of Neuroscience, Neurology Unit, Maurizio Bufalini Hospital, AUSL Romagna, Cesena, Italy; 3 Department of Biomedical and Neuromotor Sciences (DIBINEM), University of Bologna, Bologna, Italy; 4 Department of Cardiovascular Diseases, Division of Cardiology, S. Maria delle Croci Hospital, AUSL Romagna, Ravenna, Italy; 5 Department of Stroke and Neuroscience, Charing Cross Hospital, Imperial College London, NHS Healthcare Trust, London, UK; 6 Department of Brain Sciences, Imperial College London, London, UK; 7 Department of Neurology, Martin-Luther-University Halle-Wittenberg, Halle (Saale), Germany; 8 Department of Biotechnological and Applied Clinical Sciences, University of L’Aquila, L’Aquila, Italy; 9 Department of Neuroscience, Neurology Unit, S. Maria Delle Croci Hospital, AUSL Romagna, Viale Vincenzo Randi 5, 48121 Ravenna, Italy; 10 Present Address: Department of Medical and Surgical Sciences, University of Bologna, Bologna, Italy

**Keywords:** Stroke, Hyperglycemia, Hyperosmolarity, Nonketotic hyperglycemic hyperosmolar state, Stroke mimic, Neurological deficits

## Abstract

**Introduction:**

Nonketotic hyperglycemic hyperosmolar state (NKHHS) is associated with a wide spectrum of neurological syndromes including acute stroke-like deficits. Clinical features and etiology have not been established yet.

**Methods:**

Here we provide a case illustration and systematic review on non-epileptic acute neurological deficits in NKHSS. The systematic literature search followed PRISMA guidelines and a predefined protocol, including cases of NKHSS with acute stroke-like presentation.

**Results:**

The database search yielded 18 cases. Hemianopia was the most common clinical presentation (73%), followed by partial or total anterior circulation syndrome (26%). Patients with symptoms of acute anterior circulation infarct were significantly older (69.5 ± 5.1 vs. 52.2 ± 13.9 years; *p* = 0.03) and showed higher mean glucose levels at the admission vs. those with hemianopia (674.8 ± 197.2 vs. 529.4 ± 190.8 mg/dL; *p* = 0.16). Brain MRI was performed in 89% of patients, resulting abnormal in 71% of them, especially hemianopic (91%). Subcortical hypointensities in T2-FLAIR MR sequences were present in all the analyzed cases. Cortical DWI hyperintensities were also common (64%). EEG showed diffuse or focal slow wave activity in 68% of patients, especially with visual hallucinations (85%). Neurological symptoms completely resolved in 78% of patients within 6 (IQR 3–10) days, following aggressive treatment and glucose normalization.

**Conclusions:**

Our results suggest neuronal dysfunction on a metabolic basis as the leading cause of acute neurological deficits in NKHHS. Despite the generally favorable prognosis, prompt identification and aggressive treatment are crucial to avoid irreversible damage. Larger cohort studies are needed to confirm our findings.

## Introduction

Nonketotic hyperglycemic hyperosmolar state (NKHHS) is a life-threatening condition characterized by severe hyperglycemia, hyperosmolarity, and dehydration, without significant ketoacidosis. Despite a low estimated incidence, NKHHS accounts for 1% of all hospital admissions in patients with diabetes, and carries an overall mortality rate of up to 20% [1]. NKHHS results from an imbalance between insulin demand and availability, with enough insulin to inhibit free fatty acid mobilization and ketogenesis, but not enough to enhance glucose transport into cells. Severe hyperglycemia with glycosuria in turn provokes osmotic diuresis and progressive glial dehydration [2]. Neurological manifestations associated with NKHHS encompass a wide range of syndromes, spanning from seizures (15–20% of cases, usually focal motor) to hyperglycemic nonketotic hemichorea (“*diabetic striatopathy*”) and alterated levels of consciousness [2, 3]. Rarely, NKHHS can present with acute neurological deficits mimicking stroke [4]. The pathophysiologic mechanism of negative symptoms during NKHHS is still unclear. An ictal or post-ictal phenomenon (Todd’s palsy-like) has been speculated [5]. It has been suggested that a metabolic disruption of neuronal function could lead to the occurrence of neurological deficits in patients with NKHHS. This hypothesis is supported by many pieces of evidence, including the occurrence of neurological deficits even in patients with normal electroencephalogram (EEG), rarely persisting after NKHHS resolution [6–8], and the identification of peculiar magnetic resonance imaging (MRI) alterations [9].

Here we report an illustrative case of NKHHS presenting with reversible total anterior circulation infarct syndrome (OCSP-TACI, according to Bamford classification [10]), without evidence of underlying stroke or epilepsy. We also provide a systematic review of the current available literature on acute stroke-like deficits in NKHHS, in order to inform clinicians on peculiar features and work-up findings.

## Case presentation

**Fig. 1:**
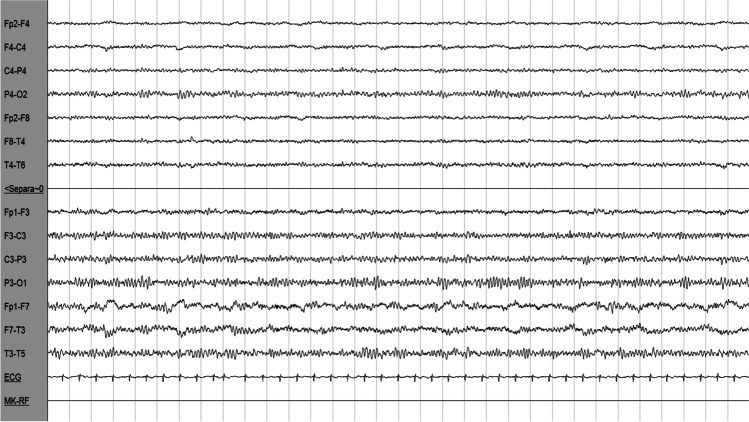
Electroencephalography (EEG). Right hemisphere slow wave activity without epileptic abnormalities (P2 montage with electrocardiography, 1.5 cm/s speed, 7.0 uV/mm sensitivity, 70.0 Hz frequency)

**Fig. 2:**
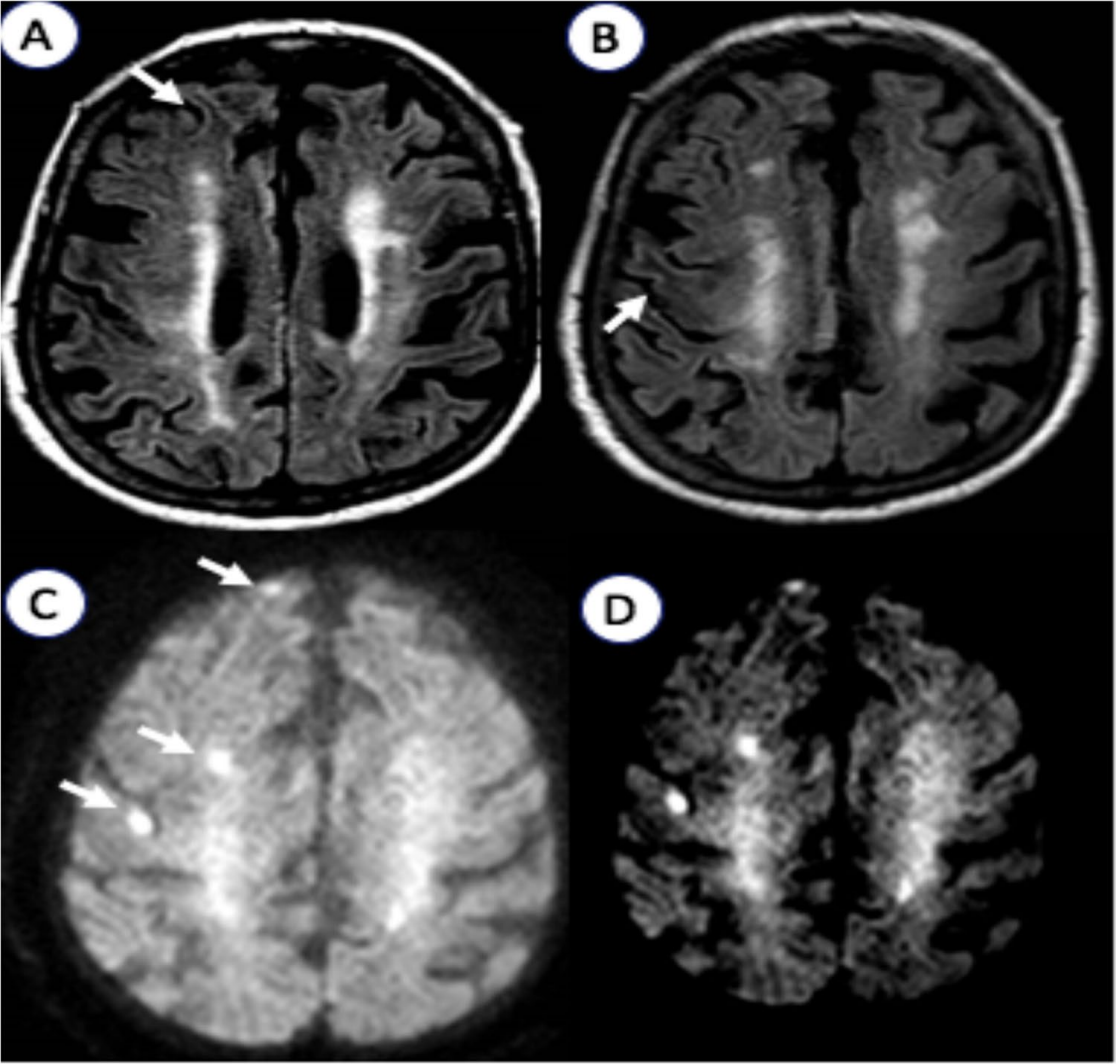
Brain magnetic resonance imaging (MRI). **A**, **B** Multifocal right hemispheric subcortical T2-fluid attenuated recovery (FLAIR) hypointensities and **C** disseminated hyperintensities on diffusion weight imaging (DWI) with **D** restricted apparent diffusion coefficient (ADC)

**Fig. 3:**
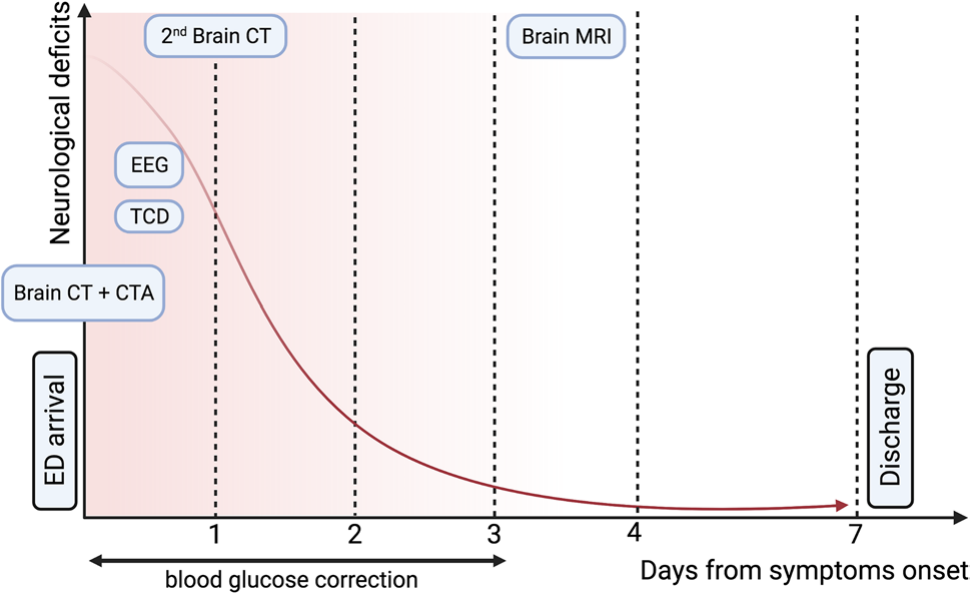
Events and investigation timeline. *Abbreviations*: CT computed tomography; CTA computed tomography angiography; ED emergency department; EEG electroencephalography; MRI magnetic resonance imaging; TCD transcranial Doppler

A 77-year-old woman with grade III obesity (body mass index 41 kg/m^2^) and a past medical history of poorly controlled adult-onset diabetes mellitus (AODM), hypertension, and hyperlipidemia was referred to our emergency department (ED) for the acute onset of left limb weakness shortly after the awakening (at about 6:00 AM). The neurological examination (9:00 AM) illustrated mild drowsiness, left side severe hemiparesis and sensory loss, left homonymous hemianopia, right gaze deviation, and dysarthria (National Institutes of Health Stroke Scale, NIHSS 27). These findings were consistent with a total anterior circulation infarct syndrome (TACI). Her blood pressure was 220/110 mmHg and did not decrease despite repeated administration of IV alpha blockers. A brain computed tomography (CT) was unremarkable for ischemic/hemorrhagic lesions and a CT angiogram excluded large vessel occlusions. Severe hyperglycemia (524 mg/dL) was found. Considering her pre-morbid functional status (modified Rankin Scale—mRS 3 for severe obesity with orthopedic comorbidity) and the elevated blood pressure and glucose levels, the patient was excluded from urgent revascularization with intravenous thrombolysis. Medical treatment was immediately started with intravenously hydration and 24-h infusion of urapidil and insulin. On day 1, the patient became less somnolent and complained of seeing flickering lights and criticized well-structured images (e.g., her daughter) appearing on her left visual field, which was normal when tested. A second brain CT was still unremarkable, while an EEG demonstrated right hemispheric slow wave activity, without epileptiform discharges (Fig. 1). A transcranial Doppler (TCD) was unremarkable. Over days 2 and 3, paralleling normalization of blood glucose levels, the patient gradually started to move her left limbs. Right gaze paralysis slowly resolved. Brain MRI performed at day 3, when neurological examination was normal, revealed the presence of disseminated right frontal cortical lesions with increased signal on diffusion-weighted imaging (DWI) sequences and reduced apparent diffusion coefficient (ADC). T2-weighted imaging and fluid-attenuated inversion recovery (FLAIR) showed ipsilateral subcortical hypointensities (Fig. 2). These alterations suggested an injury of the anterior cerebral regions induced by severe hyperglycemia [6]. Three days after symptom onset, her neurological status completely normalized (NIHSS 0). She was discharged a week after being admitted, without any neurological deficit. The timeline of events and significant investigations is shown in Fig. 3.

## Review methods

### Search strategy

The methods and guidelines of this study-level meta-analysis followed Preferred Reporting Items for Systematic Reviews and Meta-Analyses (PRISMA) guidelines and a predefined protocol shared among authors. Two reviewers (SR, MF) systematically searched PubMed for studies reporting on NKHHS and focal neurological deficits from database opening to January 2022. The search strategy was based on combination of terms, including “hyperosmolar hyperglycemic stat*,” “hyperglycemic coma”, “hyperglycemic hypersomoloar stat*”, “hyperglycemic hyperosmolar nonketotic coma”, “non ketotic coma”, and “stroke” or “neurological deficit” as either keywords or MeSH terms. Reference lists and citing articles were also reviewed to increase the identification of relevant studies. We limited the search to humans and to articles in English language.

### Selection criteria, data analysis, and presentation

We selected reports of patients presenting with NKHHS and ≥ 1 acute neurological deficit, defined as the acute onset of ≥ 1 symptom or sign in which causation can be localized to an anatomic site in the central nervous system. In order to exclude patients with epileptic negative symptoms, we included only patients who underwent at least an EEG in concomitance to focal symptoms, without evidence of congruous epileptic activity. Case selection and data extraction were performed by two authors (SR, MF) following a predefined data extraction form. The following variables were systematically collected: (1) history of AODM, (2) report of positive (e.g., hallucinations) and/or negative (focal neurological deficits) symptoms associated with NKHHS, (3) blood pressure (BP) values at the admission, (4) serum levels of hosmolarity, glucose, and glycated albumin (HbA1c), (5) timing (days from symptoms onset) and results of neuroradiological (brain MRI, MR spectroscopy, perfusion imaging) and neurophysiological (EEG) investigations. When available, clinical (degree of recovery, symptoms duration) and MRI outcomes were also collected. According to distribution, continuous data were presented as mean ± standard deviation (SD) or as median with interquartile range (IQR), whereas categorical variables as percentage. For comparing continuous variables, depending on the data distribution and the number of groups, we applied the Mann–Whitney *U* test or the *t*-test. Data analysis was made using Excel 2021 and SPSS statistical package, version 27.0 (SPSS Inc., Chicago; III., USA).

## Results

**Fig. 4:**
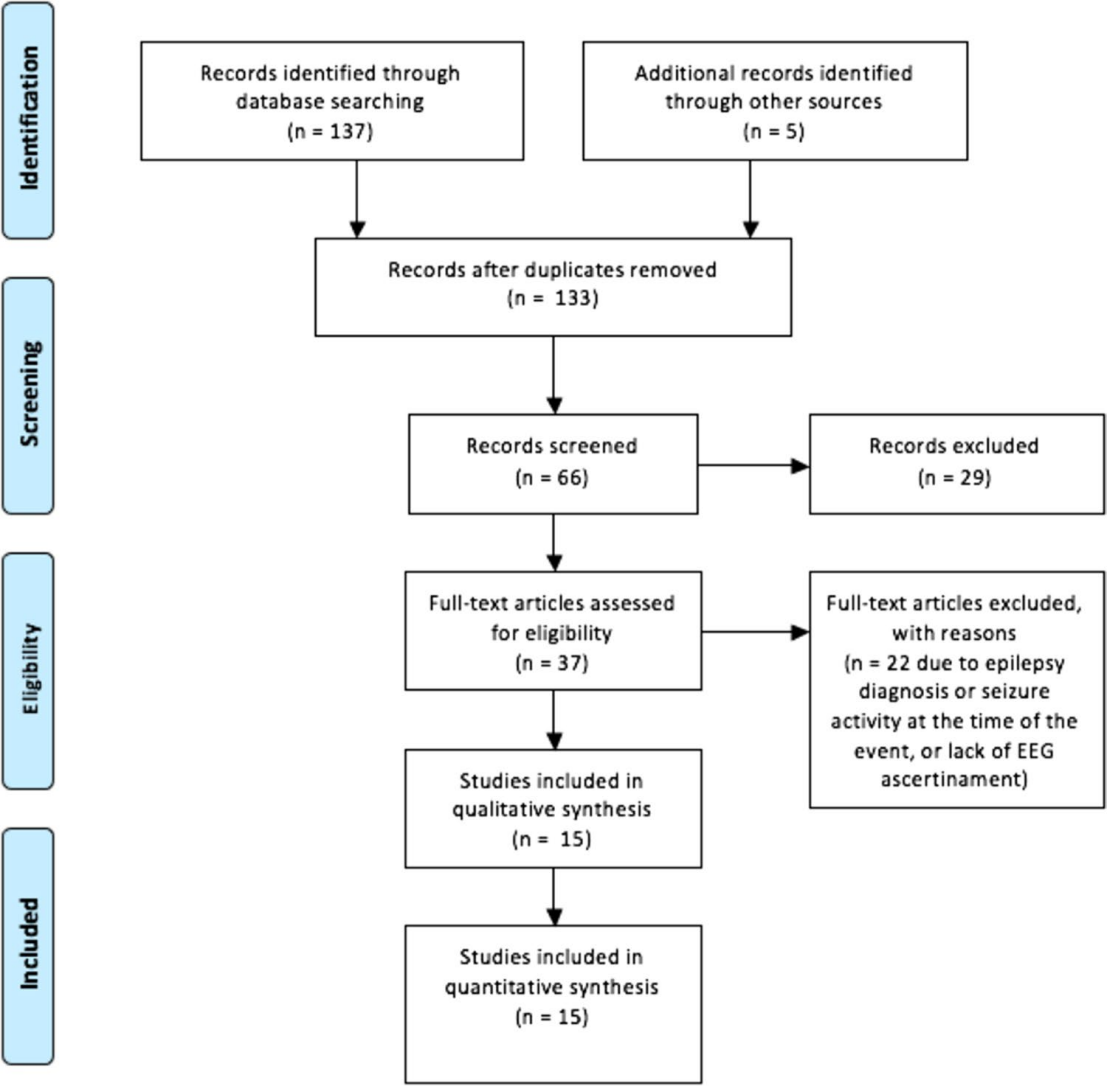
PRISMA flowchart

We identified a total of 37 articles published from 1968 to 2021 (Fig. 4), including 49 patients with NKHHS and ≥ 1 acute neurological defect. Thirty-one patients from 22 articles were excluded for concomitant epileptic activity at the EEG (*n* = 16) or for its unavailability (*n* = 15). Overall, 19 patients (18 from 15 articles [3, 6–20] and our case) were included in the analysis (Table 1).

**Table 1:** Clinical, laboratory, MRI, and EEG findings

Author (year)	Age	Sex	History of DM (type)	Negative symptoms	Positive symptoms	Glucose serum levels (mg/dL)	HbA1c (mmol/mol)	Serum hosmolarity (mOsm/L)	Blood pressure at the admission (SBP/DBP)	MRI (timing: *days after symptom onset*)	Perfusion (technique)	EEG (timing: *days after symptom onset*)	Outcome (symptom duration)
Our patient (2021)	77	F	Yes (AODM)	Right TACI syndrome	Visual hallucinations	542	93	303	↑ SBP and DBP (220/110 mmHg)	↑DWI, ↓ADC, ↓FLAIR (2 days) Follow-up: NA	Negative (CT)	Right hemisphere slow wave activity (2 days)	Complete recovery (3 days)
Kobayashi (2021) [7]	68	M	Yes (AODM)	Left hemianopia	No	524	170	NA	↑DBP (136/96 mmHg)	↑DWI, ↓FLAIR (30 days) MRS: ↓NAA, ↑choline levels Follow-up: NA	NA	Normal (30 days)	Partial recovery
Xiang et al. (2021) [14]	54	M	No	Right hemianopia	Visual hallucinations	645	134	297	NA	↓ADC, ↓FLAIR, mild PCA stenosis (7 days) Follow-up (1.5 years): temporal-occipital atrophy	Lesional hypoperfusion (MRI)	Diffuse (bilateral) slow wave activity (7 days)	Complete recovery (NA)
Da Paz et al. (2021) [12]	68	M	Yes (AODM)	Left TACI syndrome	No	600	NA	NA	NA	Normal (≤ 1 day)	↑Time to peak and normal blood volume (ischemic penumbra) of entire left hemisphere (CT)	Left hemisphere slow ways activity (NA)	Complete recovery (3 days)
Kashani et al. (2020) [20]	53	M	No	Left hemianopia	No	630	132	322	↑ SBP (150/84 mmHg)	Normal (NA)	NA	Normal (NA)	Complete recovery (7 days)
Legua Koc et al. (2020) [18]	66	M	Yes (AODM)	Left PACI syndrome	No	936	121	331	↑ SBP and DBP (181/108 mmHg)	Normal (NA)	NA	Normal (NA)	Complete recovery (2 days)
Lee et al. (2020) [19]	59	M	No	Left hemianopia	Visual hallucinations	307	121	307	NA	↓FLAIR, Gd + (NA) Follow-up (1 month): decreased gyral swelling and Gd enhancement	NA	Normal (NA)	Complete recovery (30 days)
Bala et al. (2020) [15]	55	M	Yes (AODM)	Right hemianopia	No	497	NA	NA	NA	↑DWI, ↓ADC, ↓FLAIR (NA) Follow-up (3 months): normal	NA	Normal (3 days)	Complete recovery (NA)
Lee et al. (2016) [17]	45	M	Yes (AODM)	Left PACI syndrome	No	471	149	312	↑ SBP and DBP (170/99 mmHg)	Normal (5 days)	NA	Diffuse (bilateral) slow wave activity (5 days)	Complete recovery (2 days)
Strowd et al. (2014) [6]	32	F	Yes (AODM)	Left hemianopia	No	541	124	NA	↑ SBP (160/89 mmHg)	↑ DWI, normal ADC, ↓FLAIR, Gd + (≤ 1 day) Follow-up: NA	NA	Rhythmic right temporal slow wave activity (1 day)	Persistence of left hemianopia (evolution in temporal-occipital atrophy)
	41	F	Yes (AODM)	Left hemianopia	No	306	108	NA	↑ SBP (144/67 mmHg)	↑DWI, ↓ADC, ↓FLAIR, Gd + (NA) Follow-up: NA	NA	Left hemisphere slow wave activity (NA)	Complete recovery (10 days)
Shah et al. (2014) [13]	67	F	Yes (AODM)	Left PACI syndrome	No	825	NA	NA	NA	Normal (1 day)	Small area with ↑time to peak, ↑mean transit time, and normal blood volume (ischemic penumbra) in the ipsilateral hemisphere (CT)	Left hemisphere slow wave activity (1 day)	Complete recovery (1 day)
Guez et al. (2014) [11]	61	F	No	Left hemianopia	No	943	NA	341	Normal (130/50 mmHg)	↑DWI, ↓ADC, ↓FLAIR (5 day) MRS: ↑cerebral metabolites; no changes in lipids, lactate, glucose, or ketone peaks Follow-up (3 weeks): persistence of mild occipital FLAIR hyperintensities	NA	Right hemisphere slow wave activity (6 days)	Complete recovery (3 days)
Raghavendra et al. (2007) [9]	42	F	Yes (AODM)	Right hemianopia	Visual hallucinations, focal temporal seizures	324	NA	317	NA	↓FLAIR (NA) Follow-up (3 years): mild left peritrigonal white matter loss	NA	Right parieto-temporal slow wave activity (NA)	Complete recovery (10 days)
	45	M	Yes (AODM)	Right hemianopia	Focal motor seizures	314	NA	312	NA	↑DWI, ↓FLAIR, Gd + (NA) Follow-up (6 weeks): focal cortical gliosis	NA	Normal (NA)	Complete recovery (1 day)
Lavin et al. (2005) [8]	39	M	No	Left hemianopia	Visual hallucinations	503	NA	313	NA	↑DWI, ↓FLAIR, Gd + (NA) Follow-up: NA	NA	Left hemisphere slow wave activity (1 day)	Complete recovery (3 days)
	34	M	Yes (AODM)	Right hemianopia + hemineglect	Focal motor seizures	427	NA	300	NA	↑DWI, ↓FLAIR, Gd + (NA) Follow-up: NA	NA	Left hemisphere slow wave activity (7 days)	Partial recovery (persistence of difficulty in distinguishing colors)
Brazis et al. (2000) [16]	75	M	No	Left hemianopia	Visual hallucinations	701	NA	NA	NA	NA	NA	Diffuse (bilateral) slow wave activity (3 days)	Complete recovery (2 days)
Maccario et al. (1968) [3]	73	M	No	Left PACI syndrome + right hemianopia	No	750	NA	399	Normal (100/80 mmHg)	NA	NA	Diffuse (bilateral) slow wave activity (21 days)	Complete recovery (3 days)

Abbreviations: *ADC* apparent diffusion coefficient; *AODM* adult-onset diabetes mellitus; *CT* computed tomography; *DBP* diastolic blood pressure; *DWI* diffusion-weighted imaging; *EEG* electroencephalography; *F* female; *FLAIR* fluid-attenuated inversion recovery; *Gd* gadolinium; *HbA1c* glycated albumin; *M* male; *MRI* magnetic resonance imaging; *MRS* magnetic resonance spectroscopy; *NA* not available; *PACI/TACI* partial/total anterior circulation infarct, according to Bamford et al. [10]; *SBP* systolic blood pressure; *TCD* transcranial Doppler

### Demographic, blood pressure, and laboratory findings

The mean age of patients was 54.0 ± 14.4 years. There were 11 males (57.9%) and 8 females (42.1%). History of AODM was present in 11 cases (57.9%), while NKHHS was the presenting condition of unknown diabetes in 8 cases (42.1%). BP values at the admission were reported in 9 out of 19 patients, resulting increased (≥ 140/90 mmHg) in the majority of them (77.8%). Mean values of systolic and diastolic BP were 154 ± 37 mmHg and 86 ± 20 mmHg, respectively. Mean blood glucose levels at the presentation were 567.2 ± 198.1 mg/dL, ranging from 306 to 943 mg/dL. Mean serum levels of glycated albumin were 125.8 ± 22.3 mmol/mol. Mean plasma osmolarity resulted 321.2 ± 27.6 mOsm/L.

### Clinical characteristics

Hemianopia was the most common neurological deficit on presentation (73%), and it was the sole symptom in 63% of the patients. In 2 cases, it was associated with other neurological defects, reflecting a more extensive involvement of the ipsilateral hemisphere (neglect, hemiparesis, and aphasia). Other presentations included anterior circulation infarct syndrome (26%), either complete (16%) or partial (10%). Among these patients, speech disturbances (aphasia and/or dysarthria) were the most common symptom (100% of cases with partial or total anterior circulation infarct syndrome). Positive symptoms included visual hallucination (36%), which were concomitant to hemianopia in all but our case. Four patients (21%) developed focal seizures, more often motor (75%) and always congruous with the involved hemisphere. Subjects with anterior circulation involvement were older (69.5 ± 5.1 years vs. 52.2 ± 13.9 years; *p* = 0.03) and exhibited slightly higher mean blood glucose levels (674.8 ± 197.2 mg/dL vs. 529.4 ± 190.8 mg/dL; *p* = 0.16). In most patients (78%), neurological defects completely resolved within a median of 6 days (IQR 3–10). Partial recovery with visual sequel was documented in only 4 patients (21%) who presented with hemianopia.

### Neuroradiological findings

The head CT performed on admission resulted unremarkable in all cases. A brain MRI was performed in 89% (*n* = 17) of patients, showing abnormalities in 12 (71%) of them, while it was normal in 5 cases (29%). The most common alteration consisted in focal T2-FLAIR subcortical hypointensities that were present in all patients with abnormal brain MRI. Cortical lesions with increased DWI signal were present in 9 out of 14 patients with available DWI sequences (64%) and reduced ADC in 5 out of 11 patients with available ADC maps (45%). Gadolinium (Gd) was administrated in 11 patients (64%), showing focal enhancement of the overlaying cortical gyri in 6 (55%), all with posterior involvement. Of note, only one patient with symptoms consistent with TACI syndrome showed abnormal MRI findings (1/5, 20%), whereas almost all of the patients with hemianopia showed at least one of the abovementioned MRI findings (11/12, 91%). MR spectroscopy was performed in 2 patients, showing significant increase in cerebral metabolites (creatine, choline, and myoinositol) with no changes in the lipid, lactate, glucose, or ketone peaks [7, 11]. As concern CT perfusion studies, they were performed soon after the admission in 2 cases [12, 13] presenting with TACI syndrome, showing findings consistent with ischemic penumbra (increased time to peak and mean transit time with normal blood volume). In another report, perfusion MRI was conducted about 7 days from the onset of hemianopia, revealing focal hypoperfusion in the territory of mildly stenotic posterior cerebral artery (PCA) [14]. TCD findings included mild increase in the left middle cerebral artery (MCA) mean flow velocity with reduced vasomotor reserve in a patient with right hemianopia [6], while in our case we found no abnormalities in TCD flow metrics.

A follow-up MRI was performed in 6 out 17 patients (35%) with a highly variable timespan from symptom onset (3 weeks–3 years). Total or partial resolution of MRI abnormalities was observed in the majority of cases (67%), while 2 patients (33%) developed irreversible cortical or white matter loss [9, 14].

### EEG findings

EEG resulted abnormal in 68% of patients, showing congruous focal (hemispheric or localized) slow wave activity in 69% of cases and diffuse (bilateral) slow wave activity in the remaining 31%. EEG abnormalities occurred with higher prevalence (80%) in patients with anterior than posterior circulation involvement (69%) and were frequent in patients with VHs (6/7, 85%). Serum glucose levels were similar between patients with normal and abnormal EEG findings (582.9 ± 188.1 mg/dL vs. 536.8 ± 260.8 mg/dL; *p* = 0.98). Patients with diffuse slow wave activity showed a trend to have higher glucose levels as compared to patients with focal slow wave activity (698.7 ± 52.3 vs. 553.2 ± 213.5 mg/dL; *p* = 0.09).

## Discussion

We reported the case of a patient presenting with NKHHS and clinical symptoms mimicking acute TACI syndrome, which resolved completely after the correction of hyperglicemia. Besides the very high levels of blood glucose and osmolarity, key findings of our case included the observation of MRI alterations suggestive for NKHHS and the absence of epileptic abnormalities at the EEG. Given the clinical presentation resembling acute ischemic stroke with negative brain CT, the urgent diagnosis of NKHHS is often challenging, but it should always be considered in patients with either unknown or poorly controlled AODM.

**Fig. 5:**
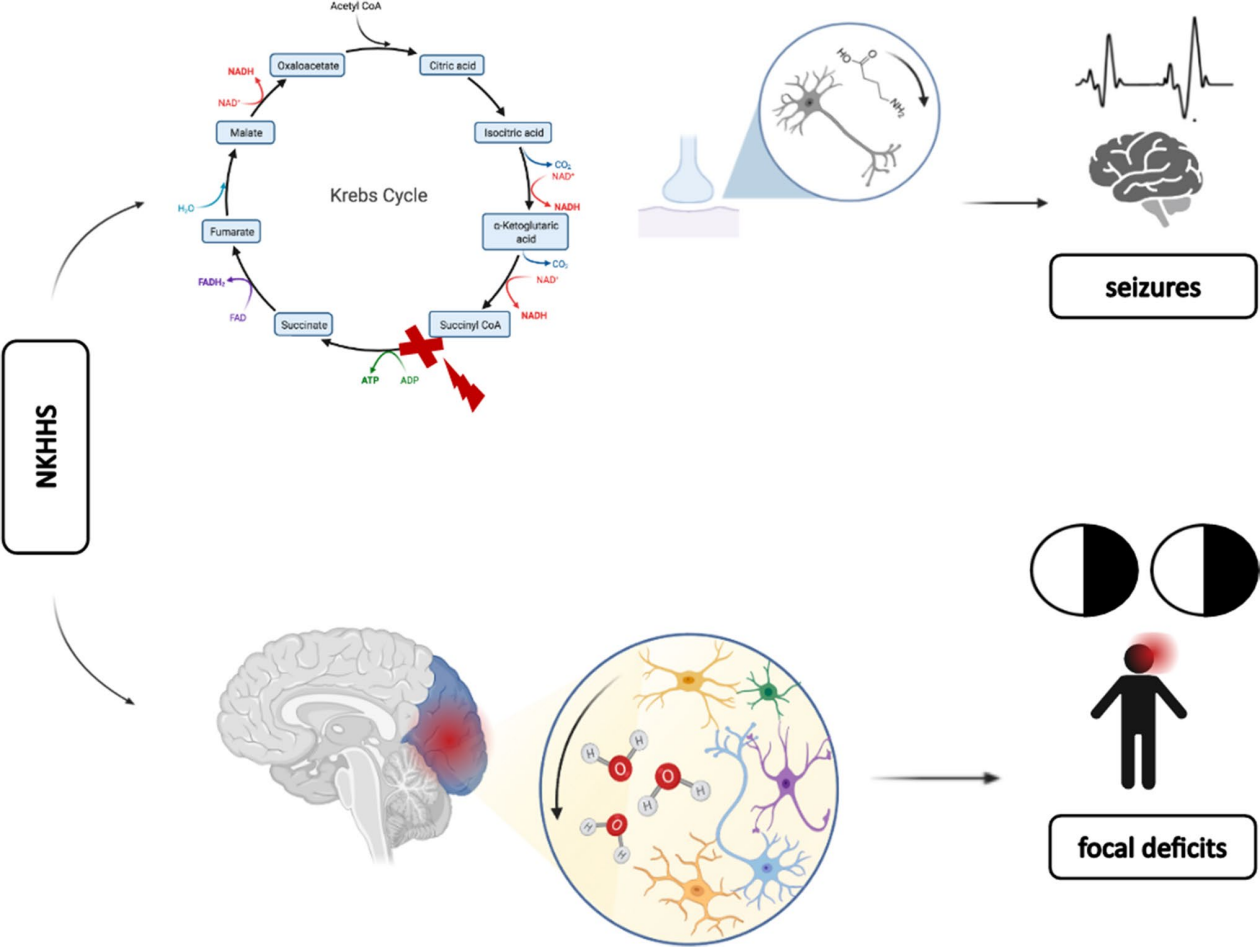
Suggested mechanisms leading to neurological symptoms in nonketotic hyperglycemic hyperosmolar state (NKHHS). Positive symptoms (e.g., seizures) may result from neuronal depletion of gamma aminobutyric acid (GABA), secondary to Kreb’s cycle disruption (GABA shunt). Negative symptoms, such as focal deficits (e.g., hemianopia), may be related to glial dehydration due to hyperosmolarity, enhancing metabolic or ischemic disruption of neuronal function

The etiology of negative neurological symptoms in patients with NKHHS is still debated. In particular, both ischemic and metabolic pathogeneses have been hypothesized (Fig. 5). Pivotal studies on animal models showed that both chronic and acute hyperglycemia may provoke a global decrease in cerebral blood flow (CBF), by enhancing focal or diffuse vasoconstriction [21]. This hypothesis is supported by the observation of increased peak systolic and mean flow velocities [22–24], along with impaired cerebral autoregulation [25], in patients with diabetes or acute glucose exposure. However, perfusion or TCD studies have not been systematically performed in patients with NKHHS-related neurological manifestations. In our analysis, brain perfusion CT/MRI showed findings consistent with ischemic penumbra in congruous brain areas of 3 patients, while in a single case TCD disclosed a mild increase of the MCA mean flow velocity [6]. Anyway, in our case TCD was unremarkable, suggesting that neuronal impairment could not be explained by a pure hemodynamic basis.

Alternatively, a metabolic etiology is suggested by the frequent finding of peculiar MRI abnormalities [9]. These include the presence of subcortical T2-FLAIR hypointensities, which represent the most frequent alteration (100% of patients), and of disseminated cortical lesions with increased DWI signal (64%) and restricted ADC. T2-FLAIR hypointensities have been related to free radical damage, iron accumulation, and early cortical ischemia [26–28], which can be promoted by either hyperosmolarity or hyperglicemia. In particular, altered mechanics of intracellular enzymes, prompted by osmotic dehydration, may generate reactive oxygen species [28]. Concomitant acidosis may reduce transferrin affinity, thus increasing unbound iron deposition [28]. Increased signal on DWI sequences indicates cytotoxic edema, potentially related to ischemia, but it may also reflect osmotic damage or hyperviscosity [6]. At a metabolic level, in NKHHS, gamma aminobutyric acid (GABA) is metabolized to succinic acid via the succinic acid semialdehyde pathway (GABA shunt), in order to provide intermediates for the Kreb’s cycle and provide energy [29]. GABA is the main inhibitory transmitter in the central nervous system and its depletion has been related to “positive” symptoms in NKHHS (e.g., focal seizures [29]) (Fig. 5). The observation of dramatically increased peaks in cerebral metabolites at MRS, without changes in lactate levels (typical of ischemic stroke), suggests that diabetes-related alterations may play a major role in inducing neuronal dysfunction. However, MRS was performed during symptoms only in 2 patients with NKHHS-related focal deficits, and larger studies are needed to confirm these findings.

In our analysis, the most frequent neurological deficit was homonymous hemianopia, which was reported (alone or in association with other symptoms) in 73% of patients. This result, along with the more frequent observation of MRI abnormalities in patients with hemianopia (91%) vs. anterior involvement (20%), may reflect an intrinsic vulnerability to hyperosmolarity of posterior brain areas. Two mechanisms could account for this phenomenon: (a) a lower density of sympathetic fibers regulating vasomotricity [30] and/or (b) a higher permeability of the blood–brain barrier (BBB) [31]. Indeed, a less pronounced capacity of blood vessels to adapt their supply in response to metabolic stress may foster neuronal dysfunction by provoking transient hypo or hyperperfusion, as occurs in posterior reversible encephalopathy syndrome (PRES) [32]. Inefficient sympathetic response might be enhanced by concomitant dysautonomia, which frequently complicates diabetes mellitus [33]. Additionally, the expression of tight junctions has been demonstrated to vary across different brain areas, potentially influencing BBB endurance to hyperosmolarity [34, 35]. The observation of Gd enhancement (an MRI surrogate of BBB disruption) only in NKHHS patients with posterior involvement might support this latter mechanism.

Anterior involvement in NKHHS seems less frequent and results more often in symptoms consistent with partial anterior circulation infarct syndrome (26% of all patients with negative deficits). Speech disturbances, and especially aphasia, were the most frequent symptoms in this subgroup (100%), probably reflecting a higher metabolic demand of related brain areas [36]. We found slightly higher blood glucose levels in patients with anterior circulation involvement, whereas statistical significance was not reached. Whether this observation will be confirmed by larger cohorts, it might imply that a higher degree of metabolic disruption is necessary to determine an impairment of anterior brain areas, perhaps for more abundant collaterals or for vasomotor differences, as discussed above. Furthermore, we found that patients with symptoms suggestive of anterior circulation infarct syndrome were significantly older compared to those with hemianopia, possibly reflecting age-related differences in brain microvasculature and cerebral autoregulation [37]. Visual hallucinations (VHs) were almost constant in patients with hemianopia (100% of cases from literature). Of interest, our patient was the sole without MRI or clinical signs of posterior involvement, thus suggesting a subtle dysfunction of occipital areas.

EEG abnormalities were common (68%), especially in patients with anterior involvement, and consisted mainly in focal or diffuse slow wave activity. Reported patterns were unspecific, and tended to be more diffuse in patients with higher blood glucose, suggesting a more severe metabolic impairment of the neuronal electrogenesis. We found a high prevalence (85%) of VHs in patients with EEG evidence of slow wave activity. VHs have been associated with the presence of occipital epileptiform discharges in some cases of NKHHS [38]. However, their frequent occurrence in the hemianopic visual field of patients without concomitant epileptic abnormalities, along with their well-criticized nature, may suggest a metabolic deficit of the cortical modulatory control (functional differentiation) with consequent abnormal activation of connected occipital areas, similarly to Charles Bonnet syndrome [39].

Prognosis of NKHHS is generally favorable, with complete recovery in the majority of cases (78%), following aggressive treatment leading to glucose and hosmolarity normalization. The persistence of visual symptoms has been reported in only 4 cases, all presenting with hemianopia, abnormal EEG, and MRI findings. A patient with persisting (although improved) hemianopia developed temporal-occipital atrophy at a brain MRI performed after 1.5 year [14]. Other two patients without persisting symptoms showed focal cortical gliosis and mild white matter loss at follow-up MRI [9]. Taken together, these findings may suggest that neuronal dysfunction could revert upon correction of the metabolic trigger, or result in irreversible damage once a critical threshold is reached. However, a follow-up MRI was performed only in 35% of patients and the timespan from symptom onset was highly variable (3 weeks–3 years). Therefore, the exact evolution of cerebral injury in NKHHS remains largely unknown. Additionally, the small number of patients with symptoms persistence and altered follow-up MRI does not enable to identify any reliable predictor of irreversible damage.

Our study has some relevant limitations. First, the small review sample, reflecting the rarity of NKHHS-related acute neurological deficits, does not allow us to draw firm conclusions on the etiology and clinical picture. Second is the inclusion of data accruing only from retrospective case reports or series, with limited impact deriving from publication bias.

## Conclusions

Neurological deficits represent a rare but clinically relevant consequence of NKHHS which can raise important diagnostic and therapeutic challenges. The clinical spectrum of NKHHS encompasses symptoms mimicking more frequently posterior circulation stroke, although the involvement of anterior brain areas is not uncommon. The exact etiology of acute focal deficits in NKHHS is still to be completely characterized, but it may rely on metabolic alterations that are driven by hyperosmolarity. Brain MRI may provide suggestive findings, while EEG abnormalities are unspecific. Prognosis is generally favorable, whereas irreversible damage may develop in a minority of cases, especially with late diagnosis. Larger cohorts are needed, possibly extracted from existing AODM registries, to verify our findings. Future studies should also investigate diagnostic and prognostic predictors in NKHHS, potentially enhancing the early initiation of proper treatment and an accurate risk stratification. From this perspective, the longitudinal measurement of blood biomarkers related to neuroaxonal and astroglial damage (e.g., neurofilament light chain protein, glial fibrillary acidic protein) could help to clarify some pathogenic and prognostic aspects. To date, the achievement of an optimal glycemic control in patients with AODM remains the best strategy to prevent severe complications, including neurological symptoms associated with NKHHS.
